# Fusion of biomedical imaging studies for increased sample size and diversity: a case study of brain MRI

**DOI:** 10.3389/fradi.2024.1283392

**Published:** 2024-04-05

**Authors:** Matias Aiskovich, Eduardo Castro, Jenna M. Reinen, Shreyas Fadnavis, Anushree Mehta, Hongyang Li, Amit Dhurandhar, Guillermo A. Cecchi, Pablo Polosecki

**Affiliations:** ^1^ SilverGate Team, IBM Argentina, Buenos Aires, Argentina; ^2^ IBM T. J. Watson Research Center, Yorktown Heights, New York, NY, United States

**Keywords:** data preparation, data scarcity, biomedical machine learning, brain MRI, dataset fusion

## Abstract

Data collection, curation, and cleaning constitute a crucial phase in Machine Learning (ML) projects. In biomedical ML, it is often desirable to leverage multiple datasets to increase sample size and diversity, but this poses unique challenges, which arise from heterogeneity in study design, data descriptors, file system organization, and metadata. In this study, we present an approach to the integration of multiple brain MRI datasets with a focus on homogenization of their organization and preprocessing for ML. We use our own fusion example (approximately 84,000 images from 54,000 subjects, 12 studies, and 88 individual scanners) to illustrate and discuss the issues faced by study fusion efforts, and we examine key decisions necessary during dataset homogenization, presenting in detail a database structure flexible enough to accommodate multiple observational MRI datasets. We believe our approach can provide a basis for future similarly-minded biomedical ML projects.

## 1 Introduction

In recent years, significant progress has been made in the field of neural networks, particularly in the domain of biomedical image analysis (1). Their performance gains, however, often require large numbers of samples for training (2, 3) in accordance with increased model complexity (4).

The issue of data scarcity as an important problem in deep learning models is a well-studied concern, particularly in the medical domain (5). This scarcity is primarily attributed to data privacy requirements, which limit biomedical data sharing (5). According to Willemink et al. (6), the chief obstacle to the development of clinical implementations of AI algorithms is the availability of a large, curated, and representative training set. While compliance with ethical code and privacy regulations are a prerequisite, the existing framework for health data collection and distribution hinders researchers from fully leveraging the potential of AI algorithms (7).

In comparison to non-biomedical public image datasets (8), most biomedical imaging datasets are orders of magnitude smaller (9). Furthermore, many of these datasets are captured within specific medical facilities, resulting in data that are limited to a particular population or group of patients and acquired using a restricted range of medical equipment. Consequently, when these models are applied in different settings, significant performance issues arise due to dataset shift (10): a mismatch in the distribution between data used for training a model and data used when applying the model due to changes in acquisition conditions (11). The need for robustness against dataset shifts in light of the restricted conditions of most studies is a key reason for combining data from multiple sources from heterogeneous settings in biomedical machine learning (ML).

Sample numbers, diversity, and robustness are not the only reasons for combining data from multiple studies. Certain models become feasible with a fused dataset that are not possible using individual studies. One example would be a model that can capture brain aging through the complete human lifespan (12, 13). Most individual studies focus on specific age ranges: some more concerned with brain development, others focus on young adults, while others target older adults. More generally, combining studies with overlapping but non-identical brain ages increases the coverage of phenotypes that be modeled simultaneously.

Integrating data from diverse studies comes with unique complications. A nontrivial one is the lack of consensus on diagnostic labels. In non-biomedical computer vision datasets, the task of mapping classes from different datasets can often be accomplished straightforwardly. When addressing object detection challenges, mapping classes representing commonly recognized objects in the human domain can be a relatively simple task and even automatized (14). In contrast, the granularity of diagnostic classes may not exhibit a one-to-one correspondence across biomedical datasets, differences that might reflect a changing understanding of pathologies (15). Secondly, datasets reflect disparate study designs. For example, some datasets belong to cross-sectional studies, while others are longitudinal. This is reflected in the organizational structure of datasets, making it challenging to define a structure that can accommodate both. Moreover, even after defining a common structure to be used across datasets, making sense of the internal structure of each study is a labor-intensive effort. It implies reviewing heterogenous source dataset documentation and comparing it with included data files. With this information, decisions must be made to map the original dataset structure to the common one. Sources of potential conflict are manifold and can range from cultural differences across studies (e.g., how levels of education are conceived in different countries) to inconsistencies between the common structure and data (e.g., how to encode a diagnosis event that occurred between visits in a visit-based study). Anticipating these challenges during the design of the common structure and before ingestion of individual studies can simplify this aspect considerably.

Here we present and discuss, as an illustrative example in brain MRI, our own implementation of a massive brain imaging dataset (MBID) from the combination of multiple smaller studies. This includes designing and creating a relational database that can accommodate all the data types required, with the goal of combining diverse source datasets into a common structure/database. We further discuss many of the challenges we faced and decisions made while building this structure and mapping individual studies to it. We examine image preprocessing steps, simple quality control measures, and provide statistics of the images contained in the MBID. We comment on limitations of our effort and steps required to expand on it to accommodate other modalities or more general study designs. We hope the discussion of the issues and solutions we implemented might help guide similar efforts in the expanding field of biomedical machine learning.

## 2 Results

**Figure 1:**
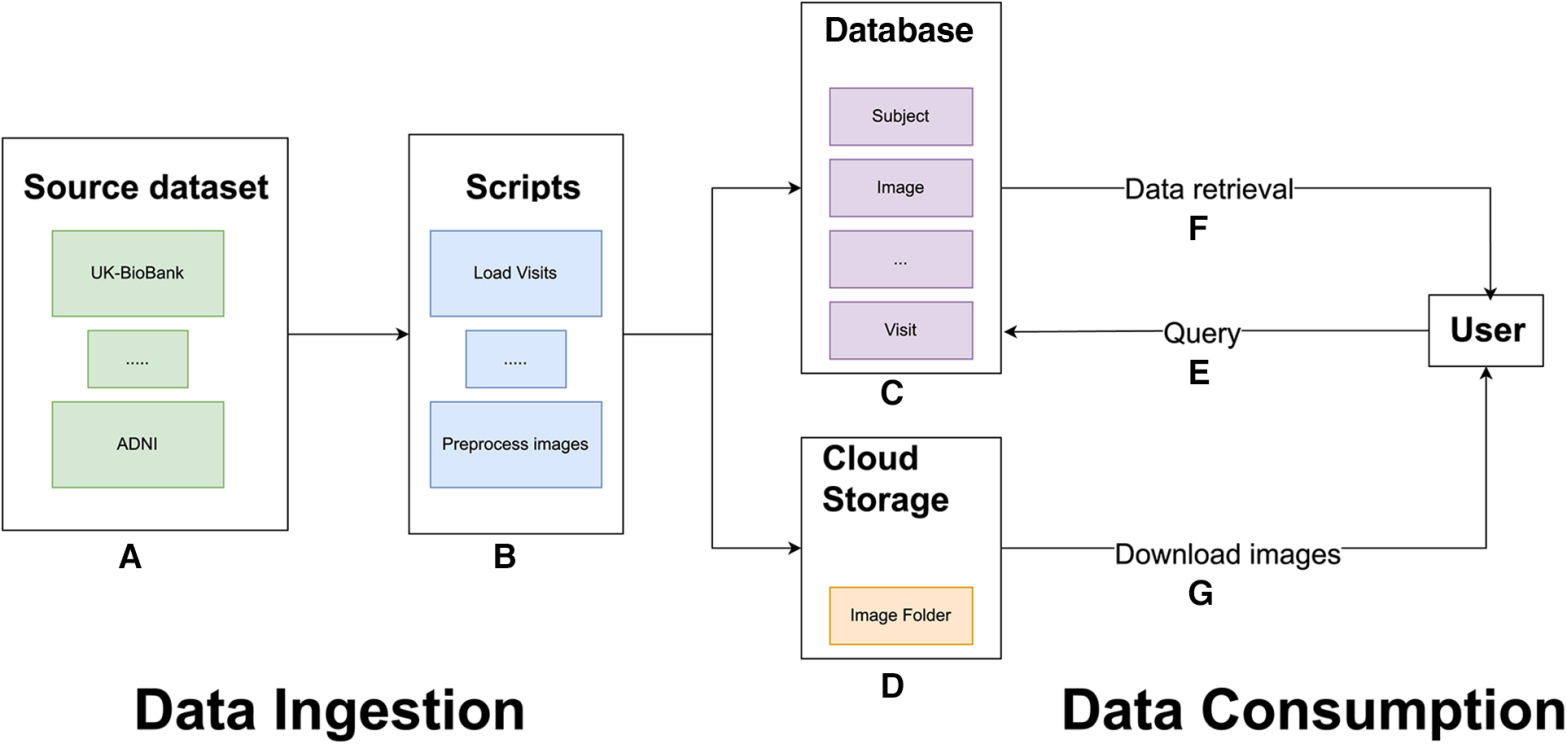
General overview of the MBID architecture. Data ingestion involves a set of source datasets (**A**) and scripts to extract, map, and load data from the source datasets into the common database and to preprocess brain images (**B**). These scripts populate a common database (**C**) and copy image files to IBM cloud object storage buckets (**D**). For data consumption, users can query the PostgreSQL database (**E**). Queries retrieve requested metadata, including image paths (**F**), which allow users to download the files from IBM cloud object storage.

To increase sample size and diversity for ML models, we built an MBID that integrates multiple studies, including large cohort studies of the general population and smaller case-control studies focusing on neurodegenerative disorders. The MBID is based on a relational database with a common structure and format to support diverse brain MRI studies. For each of the studies that we wanted to ingest, we built a study-specific set of scripts that would take as input the study data as provided by dataset sources (most commonly but not always in a CSV file), extract and homogenize information to a general format, insert those data into the common database, upload all image files to centralized IBM cloud object storage, and run a set of preprocessing steps on those images prior to applying downstream ML tasks (Figure 1). Below, we provide details about the MBID structure and discuss a number of design decisions that can be of value for similar efforts.

### 2.1 Studies used

The MBID fusion effort was based on public or semipublic observational MRI studies. Observational studies (i.e., those that take measurements in individuals without performing an intervention) are mainly divided into two categories: large cohort studies that cover the general population and case-control studies, which focus on specific conditions and tend to include fewer participants. The initial focus of the MBID was on aging and neurodegenerative disorders, so we included studies where participants were mainly adults and generally older. We plan to expand this in the future to development studies. These, however, might present their own set of challenges (e.g., for image quality controls or preprocessing). In its current version, the MBID includes 12 different studies (Table 1). They cover three different neurodegenerative disorders (Alzheimer’s Disease, Parkinson’s Disease, Huntington’s Disease) in different stages (pre-diagnostic or prodromal, and after diagnosis). The intention of combining studies covering different diseases was to support the development of large foundation models, to be validated in downstream tasks such as the detection of neurodegenerative disorders in smaller studies. The bulk of samples comes from large cohort studies, particularly UK Biobank. In terms of the total number of images, however, the combined MBID significantly surpassed the number of images provided by UK Biobank alone. However, downstream ML tasks should consider this imbalance between contributing studies.

**Table 1:** Studies included in the MBID. List of all brain MRI studies ingested in the common structure, including number of subjects and images.

Study	Subjects	Images	Population
ADNI	2,594	22,271	Alzheimer’s Disease
AIBL	703	1,310	Alzheimer’s Disease
Cam-CAN	653	653	Large Cohort
HCP-Aging	725	725	Large Cohort
Human Connectome Project	1,113	1,113	Large Cohort
NKIRS	1,267	2,564	Large Cohort
OASIS	1,088	3,358	Alzheimer’s Disease
OpenPain	117	434	Chronic Pain
PREDICT-HD	589	2,177	Huntington’s Disease
PPMI	697	1,613	Parkinson’s Disease
SALD	494	494	Large Cohort
UK Biobank	44,178	47,381	Large Cohort
**Total**	**54,218**	**84,093**	

### 2.2 MBID database

Broadly speaking, the design of the MBID involved the following steps: (1) Definition of a common database schema and entity design that could accommodate the information typically present in longitudinal and cross-sectional brain MRI datasets. This resulted in defining the entities “subject”, “visit”, “scanner”, “image”, “preprocess task”, “condition”, and “source dataset” (see Section 3 and below). (2) Manual analysis of the structure of individual studies and creation of scripts to map the source structure to the common one and its entities. The details of each of these scripts were specific to each study, as each was shared in different formats (CSV, JSON, XML) and had a specific internal structure. The idea of having a source dataset-specific script was to allow as much flexibility as possible in the ingestion profile. This could easily accommodate all the variations that we encountered, and we envision that other variations that might arise while applying the MBID to another type of use cases could be solved there. For example, if trying to ingest raw clinical images, some extra specific logic can exist in those scripts to accommodate handling duplicated reacquired images. In addition, each image was uploaded to IBM cloud storage. When consuming the common database and downloading the images from the data storage bucket, we extracted dataset subsets that combined information from multiple studies. Thus the combination of studies into a common structure produced not only a number of images significantly larger than any of the external datasets alone, but also a flexible interface for querying subsets that would be appropriate for training or testing a variety of ML models.

Because our focus was on brain MRIs, our fundamental entity was the image. As an example of how the database entities are structured, each image has the following attributes: image path, source dataset ID, visit ID, subject ID, scanner ID, and days since baseline, among others. In each “visit” entity, there is diagnosis information assigned to that visit. This allows us to use joined queries to retrieve images from a specific study or from subjects with a certain attribute (e.g., age or a specific diagnosis) or to build training and testing sets that could combine or deliberately exclude certain scanner models based on diversity considerations. Other important tables for these queries include the “subject” table, with information about subjects that would not change throughout visits, and the “visit” table, which includes information that changes longitudinally, such as the results of cognitive tests or reports of symptom intensity. Together, these provide the core tools for data subset assembly in downstream ML models.

### 2.3 Image preprocessing

**Figure 2:**
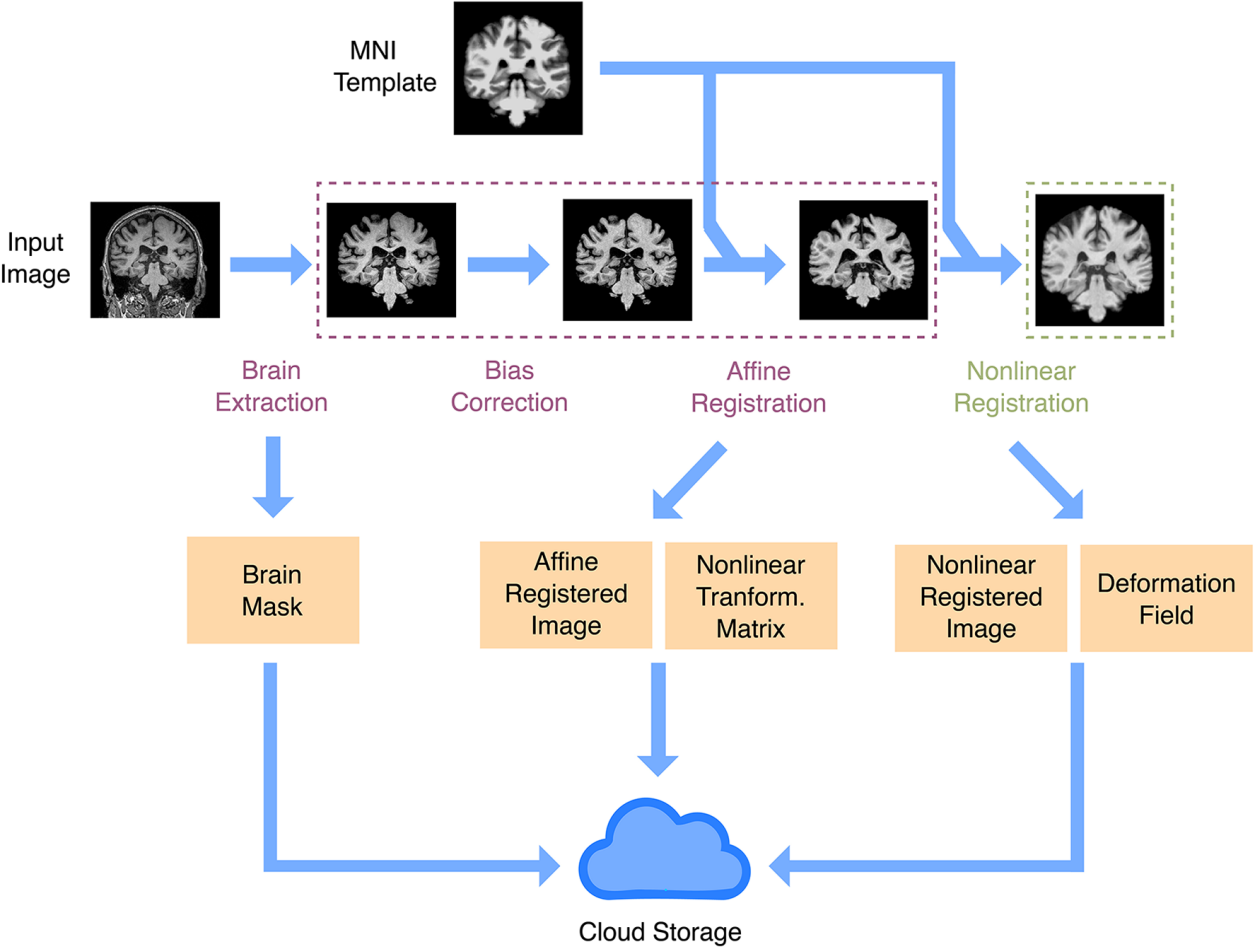
Anatomical image preprocessing. The main preprocessing goal is to have images registered in a common space. There are two registration pipelines: linear and non-linear. Both registration pipelines take a raw image and the MNI template as inputs, do brain extraction, intensity bias correction, and affine registration (rigid plus global scale parameter). In the non-linear registration pipeline, there is an extra step to produce a non-linear warp, using as input the affine registration. Outputs include a brain mask, the registered image, the affine registration matrix, and the non-linear warp. All output files are uploaded to IBM cloud object storage.

.Our structure supports different image types, such as functional, diffusion, and T1-based images, and it is straightforward to extend it to more modalities. For each desired modality, a fast preprocessing pipeline will be required for large-scale cohorts. Here, we implemented one for T1 images only. We needed a fast, lightweight pipeline that would be portable and easily installed in a remote virtual machine or a server cluster. We therefore avoided common but bigger packages such as FreeSurfer (16) and implemented simple pipelines using Python-based tools (see Section 3). Preprocessing consisted of two pipelines (Figure 2). One was a fast affine registration pipeline meant to align images to the standard MNI template while preserving their anatomy, as is recommended for CNN models in brain MRIs (17). This process usually took around 15s per sample on a single CPU. We also implemented an additional non-linear registration step. This more time-consuming step (around 75s per sample on a single CPU) warps the anatomy to the template. It is useful for more traditional univariate statistics, but also for aggregating maps from multiple samples into summary maps (e.g., feature importance maps). After running the preprocessing steps, we store the resulting files in the database using the preprocessing-related entities. Running the preprocessing on the fly will be unsuitable for most ML downstream tasks, given the number of images available. When estimating processing times in large datasets hosted remotely, it is important to take into account read/write rates, which could constitute the effective bottleneck for minimalist preprocessing.

### 2.4 Quality controls

Images can come with a myriad of quality issues. To some extent, they constitute part of the heterogeneity to which ML models are meant to be exposed by fused datasets from a large number of sites. Most studies apply some method of quality control in the images they include. Because of the extremely large number of samples considered in our study, visual quality assessments were impractical. Even automated QA tools (18) were orders of magnitude slower than the preprocessing itself. The speed of processing was especially sensitive, given the number of images contained in the MBID. This was a reason not to use more comprehensive quality control packages such as MRIQC. In addition, the relevant quality metrics depend on the downstream task: e.g., if the ultimate goal is age prediction, this should be possible even in the presence of artifacts due to eye movement. We decided to focus on assessing that images contained a relatively whole image of the brain that would reasonably overlap with the MNI template after preprocessing. We used the voxel-wise Pearson correlation coefficient of voxel intensities with those inside the MNI brain template as a simple measure of brain image integrity and registration success, which we and others have used in the past (19, 20). In downstream tasks, we found a cutoff of 0.4 reliably excluded poorly registered subjects while minimizing incorrect exclusion of normal anatomical variation. It must be highlighted, however, that other measures might be relevant, depending on the ML application and the source data ingested. If MBID is adapted to ingest raw clinical images, another type of quality control pipeline might need to be implemented, depending on the expected issues.

### 2.5 MBID: subject and image statistics after fusion

**Figure 3:**
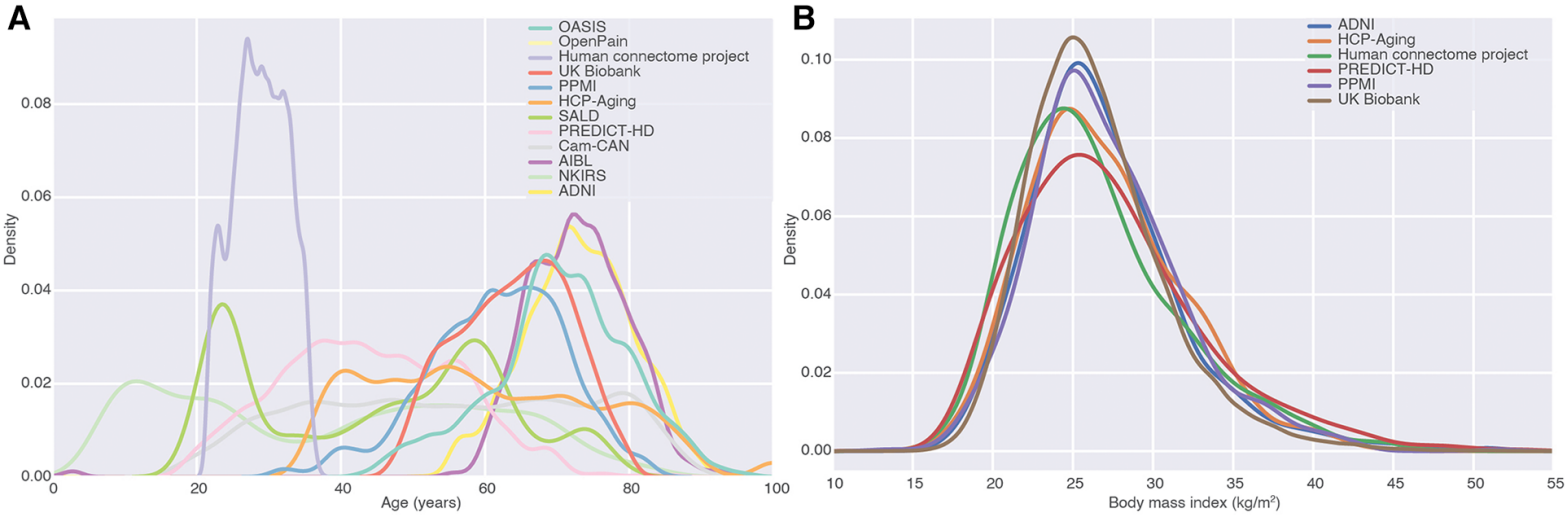
Fusion of studies as a source of phenotypic diversity. (**A**) Density of age distributions of each of the datasets in the MBID. We focused on studies covering healthy adults and elderly populations. (**B**) Body mass index distributions for different studies in the MBID. Studies with almost no overlap in one variable (age) can overlap remarkably in other ones, highlighting the richness of fused datasets for the modeling of phenotypic diversity.

After ingestion of the studies, we had 84,093 images from 54,218 subjects (Table 1). These came with a number of demographic and phenotypic information, including disease diagnoses. One of the major applications of ML in large MRI cohorts is modeling of brain aging. The resulting MBID captured a marked heterogeneity in this regard, with different studies covering different but overlapping age ranges (Figure 3A). Age was far from the only phenotypic information available. For instance, in many studies, body mass index was available, and studies overlapped remarkably in that regard, even when they covered very different age ranges. This showcases the value of the MBID for very different applications.

## 3 Methods

### 3.1 Tools

The MBID consisted of a relational database and cloud storage for images and other files. As our database engine, we utilized PostgreSQL 12.14. All database entities and relations were defined using the Python package SQLAlchemy. Python 3.7 was the primary programming language for all our code, including the scripts for ingesting external datasets. IBM Cloud Object Storage was utilized to store unstructured data such as images, CSV, or JSON files.

### 3.2 Database design

**Figure 4:**
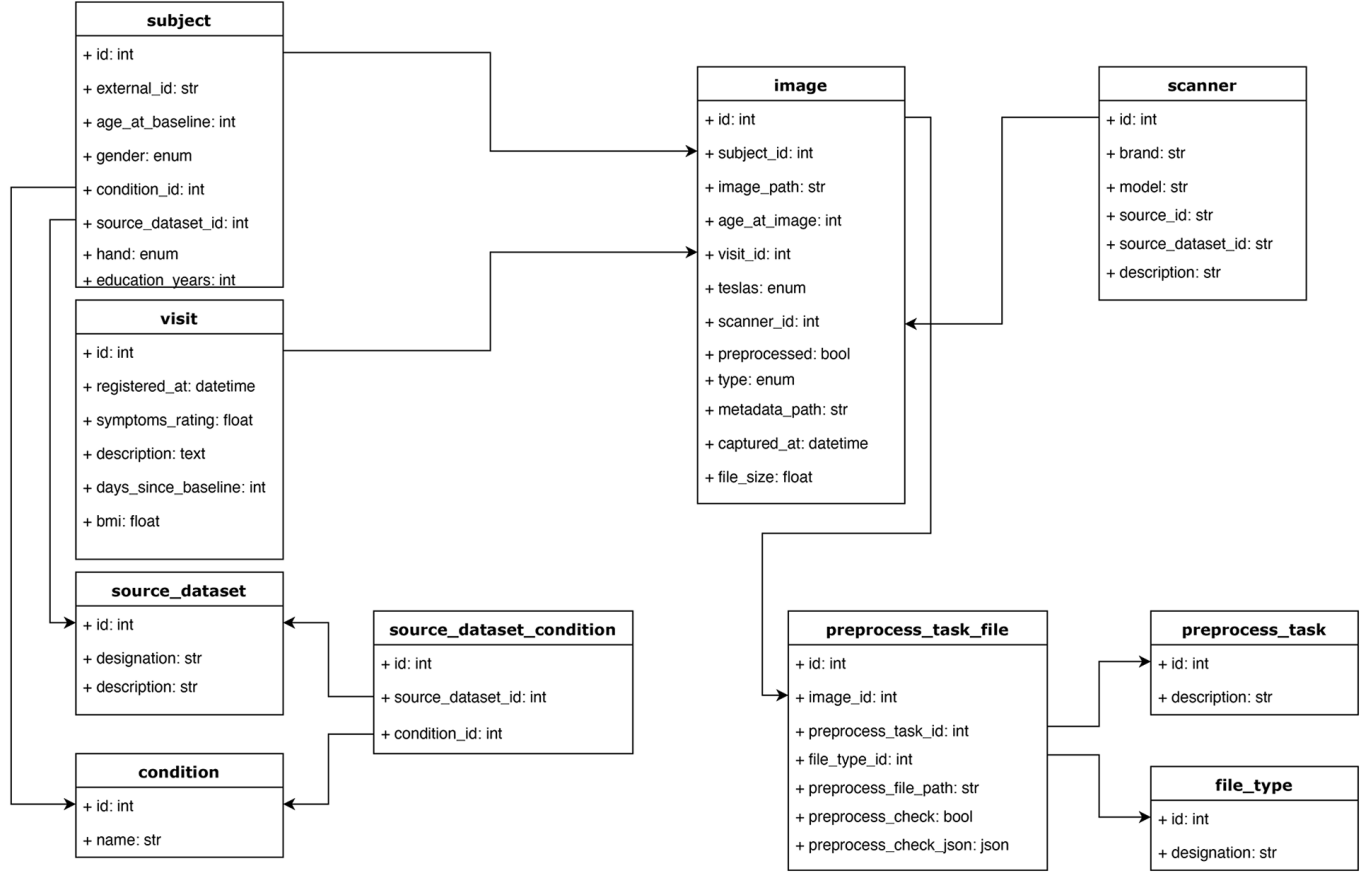
Database schema. The main objective of this structure was to accommodate the relevant information from each study, capturing it while keeping a relational structure that would allow for easy filtering and querying of the data. For example, instead of storing scanner information as a string field in the image table, we defined a scanner table, which allows querying images from different studies that were acquired with similar scanner devices based on some of the fields in the scanner table. All the tables and relations are shown.

We organized our database under the following entities (Figure 4):

• Source dataset: This entity includes the name of the dataset and serves the purpose of identifying the source study for each subject.
• Source dataset condition. This entity lists all possible clinical conditions of the participants in the studies. This attribute is encoded as an integer and present for each study.
• Condition: This entity maps the conditions (encoded by integers) to their names. Together with “source dataset condition”, this entity allows one to filter studies that contain or do not contain a certain condition. This mapping (between the source dataset condition and a unified list of conditions) was manually carried out as we focused on some key conditions in our experiments. Nonetheless, adding an extra field to the condition table and supporting a condition ontology with a coding system would be straightforward.
• Subject: This entity contains a participant’s identity code and his/her demographic information, such as gender, handedness, age at baseline (i.e., first visit on the study), education years, diagnostic condition, as well that “source dataset id”, which was the ID code of the participant in the study from which his/her data were collected.
• Visit: Most longitudinal studies are structured around clinical visits where measurements are taken and constitute longitudinal time points. This entity contains information about each visit from each participant across studies, including days since the baseline visit, a condition ID to indicate the diagnosis (if any) at that visit, body mass index, symptoms (can contain other clinical scores), and source dataset ID.
• Scanner: This entity has information about the medical devices used to acquire the images. It includes the brand, model, magnetic field strength, and source dataset ID. This entity serves, for our purposes, as a way to encode site differences. Some facilities have more than one scanner, complicating this identification between scanner and site, but we kept just a scanner entity for simplicity.
• Image: Each image is related to a “visit”. This entity includes information about the type of image (this field stores the modality of the image, such as T1-weighted or any other format that is used in the study), preprocessing status (none, affine, or non-linear), visit ID, file size, subject ID, scanner ID, image file path (in an IBM cloud object storage bucket), days since baseline (sometimes the image was acquired on a different date than its associated visit), metadata path (could be used to store a JSON file, with any type of information, this field might be especially useful if adapting the MBID to a different use case) and source dataset ID.
• Preprocess task: This entity stores the types of preprocessing supported in the pipeline. It contains a description and helps store the type of preprocessing applied to a given image in a relational way.
• Preprocess task file: This is the entity that stores the new images and other outputs generated after applying preprocessing methods to the original images. It includes the original image ID, type of preprocessing, type of file, and the path of the new file (in an IBM cloud object storage bucket). This entity can store auxiliary files used during the preprocessing task to make it reproducible and keep track of the complete pipeline.
• File type: This entity helps distinguish the resulting files from the preprocessing module, such as the registered brain image, its mask, and affine and non-linear transforms. Each set of newly created files have a file type id to allow filtering the desired one while making a query. Having this information in an entity, as opposed to just having a field in the preprocess task file, provides more flexibility, allowing the addition of new file types while creating new preprocessing tasks.

### 3.3 Pipeline for study ingestion and design decisions

**Figure 5:**
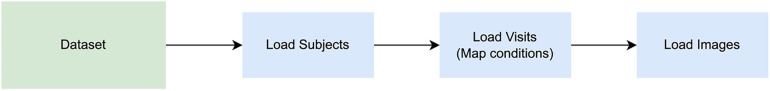
Image loading steps. A series of scripts must be run when loading a new dataset: one to populate the subjects structure, a second one for visits, and another for images. The scripts to load subjects, load visits, and load images, should be executed in said order to keep the consistency of the database tables, respecting the relationship among entities.

Each study presented unique challenges for data ingestion. The process of adding a new study consisted of (1) understanding its associated files and structure (2) creating a study-specific script in which the study data were mapped to the homogenized structure proposed in this work. For most cases, three scripts were created for each study: “Load subjects”, “Load visits”, and “Load images”, to be run in that order, as each entity requires the previous one to be consistent (Figure 5). All studies were already anonymized. The anonymization procedure was specific to each source dataset and is described in each source dataset publication cited in this work.

Image files were provided by the source studies by different methods (including but not limited to FTP servers and S3 buckets). We uploaded all files to a private IBM Cloud Object Storage, from which subsets could be downloaded as needed for ML applications. This includes the results of image preprocessing as encoded in the “preprocess task” entity of the common structure.

Even with a relatively general database structure, the diversity of the combined studies created numerous challenges. A basic one was that some of the studies are longitudinal (multiple visits) and others cross-sectional (no visit-structure). As a simple solution, we decided to associate each image with a corresponding visit in all cases, even though this concept did not exist in the source data. On the other hand, some of the studies included visits without a corresponding image, but still had useful information (such as cognitive scale results). This is what caused us to consider the “visit” entity as separate from the “image” entity, allowing visits without an associated image.

In some longitudinal studies (ADNI), the dates of images and clinical visits often differed, while the diagnostic information pertained to the “visit” entity. Consequently, matching an image with its corresponding clinical visit required a decision. Our solution was that each image would be associated with its closest visit in the past, if there was one, up to a theshold of 60 days. The rationale for this was that this would prevent assigning diagnostic conditions from an assessment that had occurred after an image was acquired, since the focus was on using images and concurrent information to make prognostic models, and it was important to avoid contamination from future events. The threshold was chosen as it matched the distribution of gaps between image acquisition and clinical evaluation and was below the general timescale of neurodegeneration.

Each study provided its own list of diagnostic conditions. To unify those conditions, we manually mapped them to a master list. This was simple and straightforward as all studies shared a similar granularity level of the diagnostic conditions and we focused on a reduced set. It would have been possible to map those conditions to an ontology, but this would have been more complex and unnecessary for our specific use case. There is a trade-off between the level of detail and complexity of the process, which must be weighted depending on the intended use case.

To mention just one of the more minor challenges, cultural differences across studies complicate their fusion. A case in point was the standardization of education by converting its total to number of years, an important demographic value, which was not readily available in all studies. One of those was UK Biobank, where instead a field specifies the type of degree obtained within the UK education system (https://biobank.ndph.ox.ac.uk/showcase/field.cgi?id=6138), which has changed over time. Thus, it was necessary to estimate the typical duration and order of each of these educational milestones. Such adaptation of the idiosyncrasies of each study is a key element of the scripts of the pipeline.

Establishing an internal folder structure for storing image files in the IBM cloud object storage solution was also necessary. One attractive format for organizing images is BIDS (21), which sets guidelines for folder names per patient and image modality. In the MBID, most metadata exist in the entities of the proposed database schema, and images were retrieved via a database query. Because of this, the MBID does not follow that folder prescription, keeping with the structure of each original study upon uploading it. It might, however, be useful as a basic for a potential data export script, in which the output format could respect BIDS guidelines.

### 3.4 Studies

The current iteration of MBID combines the following observational studies: ADNI (22), AIBL (23), Cam-CAN (24, 25), HCP-Aging (26), Human Connectome Project (27), NKIRS (28), OASIS (29), OpenPain (https://www.openpain.org), PREDICT-HD (30), PPMI (31), SALD (32), and UK Biobank (9). Appropriate user agreements were signed and its conditions were followed.

### 3.5 Preprocessing tools

Our lightweight preprocessing pipeline was based on the ANTsPy (33) and the deepbrain (https://github.com/iitzco/deepbrain) packages.

## 4 Discussion

In this work, we wanted to illustrate the challenges and benefits of data fusion in the light of our own effort in this area. We presented the challenges and decisions we faced while combining brain MRI datasets from different sources. We also explained the flexible structure we created to accommodate most brain MRI observational studies.

We combined 12 studies into a homogenized database, allowing access to more than 84,000 images from approximately 5,400 subjects, covering the adult lifespan. We combined data from 88 scanners, based on different facilities and geographical locations, likely boosting the robustness and adaptability of any ML model based on it. In keeping our approach as flexible as possible for brain MRI images, we have created a structure that should easily accommodate further brain MRI datasets and preprocessing pipelines.

There are multiple reasons for engaging in fusion efforts. As mentioned, higher sample numbers are necessary for complex models in high-dimensional feature spaces, and heterogeneity is key for robustness. Also, the gathering of studies covering different phenotypes (e.g., disorders) enable models that are would be impossible with single studies, such as modeling of aging across the whole human lifespan is one example. In addition, availability of samples with multiple diagnoses coming from different studies allows one to investigate commonalities across them, using for instance transfer learning approaches (? ). It is critical however to be mindful of the confounds that are simultaneously introduced by fused datasets. For instance, when validating a differential diagnosis approach in which samples with two disorders come from different studies, one must take steps to ensure that the model is reflecting real signatures of the disorders and not just differences in data acquisition across studies, that no harmonization technique can remove completely.

In general, for the task of homogenizing a set of biomedical data sources with different studies than the ones we presented, we suggest first analyzing the proposed entities and seeing if the data needed to be stored can be accommodated to our proposed structure. All the structures created and the types of downstream tasks covered were related to brain MRI images. If this structure is used for other kinds of biomedical images, it would likely be necessary to adapt the proposed entities for the new use case. We expect that extra information might be needed in the Image entity in a different use case (one might want to store information about contrast agents or a medical report in a more clinical setting) or in the preprocess-related entities, where the required steps might be completely different in other settings, such as in oncology. That said, we believe the general ideas of this work should help guide the creation of other homogenized biomedical databases.

It would be ideal to offer a large fused dataset publicly, as with other biomedical modalities in projects like MED-MNIST (34). In our case, and given the terms and conditions of the source datasets used in our work, this was not possible. Most studies require special agreements between the user and the organization that compiled or produced the data. It has been argued (35) that a solution for digital health issues around data privacy might be solved with federated learning, in which users send their model’s training pipeline to be run in a closed infrastructure, with access to the data compilation but without allowing users to download the data directly. This kind of solution could be implemented with the centralized data we propose in our work but would require dedicated infrastructure and further agreements with the data owners.

As a next step, it would be useful to expand the methods available to retrieve the data by implementing an API to facilitate the consumption of the MBID. This would allow more fine-grained control of data access, allowing different users to download only the datasets for which they have shared access permissions from the source dataset organization. In that scenario, it would be valuable to produce an export of the data in a format such as BIDS (21), an intuitive standardization of brain image storage that defines guidelines for folder names per patient and image modality.

One main assumption of the MBID structure is that studies have their data collected in discrete visits. There are increasingly more clinical studies that are utilizing measures outside hospital visits, such as ecological momentary assessments (EMAs) (36). A case in point is the AMP SCZ partnership in psychosis prediction (37), which combines measurements from clinical visits with a number of digital assessments, both active (e.g., daily surveys) and passive (e.g., sleep behavior, text messaging and calling behaviors). Thus, one important future direction would be extending the structure to accommodate these types of data. This might require the creation of a new set of entities to store the digital data and relate to visits in terms of days from baseline. On a different dimension, integrations with popular Electronic Health Records (EHR) (38) software could allow to populate the common database with a rich amount of observational data and provide the flexibility to ingest data generated in real time. The integration with EHRs can be accomplished using common standards such as FHIR (39). Nonetheless, each healthcare system integration will require special data privacy and anonymization considerations.

In conclusion, for the future of the biomedical ML field, it is important to find solutions to increase sample sizes and heterogeneity. Here we presented an example implementation of a fusion strategy to augment the data available, potentially applicable in a large number of settings, and illustrative of the challenges and limitations of these approaches. We believe that it can provide a flexible basis for others considering similar efforts.

## Data Availability

The original contributions presented in the study are included in the article/Supplementary Material, further inquiries can be directed to the corresponding author. The source code for the database structure and preprocessing pipelines proposed in this manuscript is available at https://github.com/IBM/MBID-db.
